# A Cross-Sectional Survey of the Association between Bilateral Topical Prostaglandin Analogue Use and Ocular Adnexal Features

**DOI:** 10.1371/journal.pone.0061638

**Published:** 2013-05-01

**Authors:** Mamta Shah, Grace Lee, Daniel R. Lefebvre, Benjamin Kronberg, Stephanie Loomis, Stacey C. Brauner, Angela Turalba, Douglas J. Rhee, Suzanne K. Freitag, Louis R. Pasquale

**Affiliations:** 1 Department of Ophthalmology, Boston University School of Medicine, Boston, Massachusetts, United States of America; 2 Massachusetts Eye and Ear Infirmary, Department of Ophthalmology, Harvard Medical School, Boston, Massachusetts, United States of America; University of Florida, United States of America

## Abstract

We studied the relation between prostaglandin analogue use and ocular adnexal features. We used a prospective, cross-sectional study involving 157 current, 15 past, and 171 never users of prostaglandin analogues. Patients 50 years of age or older and without conditions affecting ocular adnexal anatomy underwent glaucoma medication use history, external digital photography and systematic external adnexal exam. Two masked readers assessed the digital photos for upper lid dermatochalasis and lower lid steatoblepharon using a validated grading scheme. Another masked clinical examiner also assessed upper lid ptosis, levator muscle function, and inferior scleral show. We performed ordinal logistic regression analysis accounting for multiple covariates to assess the relation between prostaglandin analogue use and adnexal features. Multivariable analyses indicated there was a 230-fold increased risk of incremental involution of dermatochalasis (odds ratio (OR)  =  2.30; 95% confidence interval (CI) 1.43–3.69; p = 5.44E-04) and a 249-fold increased risk of incremental loss of lower lid steatoblepharon (OR  =  2.49; 95% CI, 1.54–4.03; p =  1.98E-04) associated with current prostaglandin analogue use (bimatoprost 0.03%, travoprost 0.005%, or latanoprost 0.004%) versus prostaglandin analogue never or past users. Upper lid ptosis (OR  =  4.04; 95% CI, 2.43–6.72; p = 7.37E-08), levator dysfunction (OR =  7.51; 95% CI, 3.39–16.65; p = 6.74E-07) and lower lid retraction (OR = 2.60; 95% CI, 1.58–4.28; p = 1.72E-04) were highly associated with current prostaglandin analogue use versus prostaglandin analogue never or past users. The associations between prostaglandin analogue use and deepening of the upper lid sulci and between prostaglandin analogue use and loss of inferior periorbital fat are confirmed in this multivariable analysis. The associations between prostaglandin analogue use and levator muscle dysfunction and between prostaglandin analogue use and upper lid ptosis represent significant side effects that could impact visual function in glaucoma patients.

## Introduction

Prostaglandin analogues (PGAs) are a class of ocular hypotensive agents that lower intraocular pressure (IOP) predominantly by enhancing uveoscleral outflow [1]. Once daily dosing of either latanoprost, bimatoprost or travoprost produce ≥20% IOP reduction in at least 70% of patients using mixed treatment comparison analysis [2]. The most common ocular adverse effects of the PGAs include periocular skin pigmentation, hypertrichosis, conjunctival hyperemia, iris color change and ocular pruritus [3]. Other less common, but fairly well established side effects of the PGAs include uveitis, cystoid macula edema, and reactivation of herpetic eye disease [3]. Observations in monocular users have revealed a novel set of ocular adnexal changes attributable to topical PGA therapy; namely, deepening of the upper eyelid sulcus (DUES), upper lid ptosis, loss of the inferior orbital fat pads, and enophthalmos, a constellation of symptoms referred to as prostaglandin-associated periorbitopathy (PAP) [4], [5]. PAP, which can be easily dismissed as age-related adnexal findings in bilateral users, has been recognized in several small case series involving both unilateral [6] and bilateral PGA users [7]. Collectively these series demonstrate that PAP was not an antecedent finding and that the adnexal changes partially reversed when the PGA was withdrawn. Furthermore evidence exists that all three members of the PGA class can be associated with PAP [8], [9].

We performed a large cross-sectional study to confirm whether PAP is clearly associated with PGA application among bilateral users using a validated grading scheme applied by masked observers and confirmed by clinical examination. We performed multivariable analyses to assess whether PAP was independently associated with PGA use or whether it was the result of confounding features such as age, ethnicity, body mass index or use of other classes of glaucoma medications. We fully categorize the nature of PAP and assess the strength of association between PGA use and adnexal changes. The latter finding provides the physician with an assessment of the extent of PAP among current users, as this information cannot be readily inferred from the previously reported case series.

## Methods

### Ethics Statement

**Figure 1:**
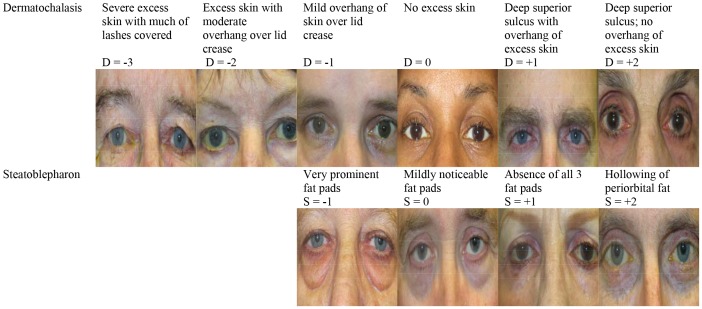
Grading Scheme for Dermatochalasis (D) & Steatoblepharon (S). Digital external photographs representing each grade of D and S for selected participants enrolled in the study.

The Human Studies Committee of Massachusetts Eye & Ear Infirmary approved this study. All participants provided written informed consent to participate and these forms are retained on file with the principal investigator (LRP). The informed consent was witnessed and cosigned by study personnel (usually MS). The Human Studies Committee of Massachusetts Eye & Ear Infirmary approved this informed consent process. Furthermore, all study participants in Figure 1 have given written informed consent, as outlined in the PLOS ONE consent form, prior to publication of their photographs.

### Description of Study Population

343 patients (186 females and 157 males) were recruited from the Glaucoma Service and Comprehensive Eye Service at Massachusetts Eye & Ear Infirmary (MEEI) over the course of seven months in 2011.

### Inclusion/Exclusion Criteria

Patients were excluded from the study if they had the following conditions that could affect ocular adnexal features: (1) history of intraocular procedures (aside from childhood strabismus surgery and prior laser surgery); (2) pregnancy [10]; (3) active adnexal infection; (4) medical conditions such as thyroid disease, Addison's disease, and congenital, myogenic, or neurogenic ptosis; (5) current use of oral corticosteroids [11] and (6) prior orbital disease or eyelid surgery. Exclusion criteria were confirmed by taking patients' history and analysis of the medical records. Patients age 50 years or older who were able to give informed consent and did not meet exclusion criteria were eligible for the study.

### Validation of a Grading Scheme for Assessment of Dermatochalasis and Steatoblepharon

We surveyed the external photographic library of an oculoplastic surgeon (SKF) to derive a grading scheme for upper eyelid dermatochalasis (D) and lower eyelid steatoblepharon, defined as inferior adnexal extraconal orbital fat herniation (S). We chose standards for each category from the library. The grading scheme for D and S centers on the value of zero (**Figure 1**), which we defined as no excess upper eyelid skin and no protrusion of the inferior orbital fat pads, respectively. Specifically, D scores ranged from −3 to +2 (−3 = severe excess skin; +2 = deep superior sulcus with absence of excess skin) while S scores ranged from −1 to +2 (−1 =  very prominent fat pads; +2 =  total hollowing of inferior periorbital fat). Subsequently, we took 40 preliminary digital photographs of glaucoma patients not included in this analysis with a Canon G12 10MP digital camera (Newport News, VA). Two masked readers graded the digital photos for D and S scores using the grading scheme described in the **Figure 1**. Initial intra- and interobserver agreement of two masked readers was assessed with the kappa statistic and found to be high for D and S scores (κ≥0.74). Once the grading scheme was established we adopted the following standardized photographing protocol: we took three external digital photographs (a frontal view and 45 degree lateral views from the right and left side) of each patient. Photographs were taken with patients seated at a distance of 24” from the camera prior to the instillation of mydriatic agents. They were instructed to face the camera directly with their eyelids open, brows relaxed, and head level with the lens of the camera.

### Clinical Examination

After measuring height and weight on each subject, a masked examiner measured the upper eyelid margin-to-corneal light reflex distance or MRD 1 (to assign a P, or ptosis score), lower lid margin-to-corneal light reflex distance or MRD 2 (to assign an I, or inferior scleral show score), and levator muscle excursion (to assign an L score) in both eyes. Furthermore the examiner assessed dermatochalasis and steatoblepharon based on the grading scheme described above and illustrated in **Figure 1**. This examination was completed prior to the instillation of dilating drops. To measure MRD1 and MRD2 the patient was requested to look at the light source (pen light) and the distances from the corneal light reflex to the upper eyelid and to the lower eyelid, respectively, were recorded. Specifically, P scores ranged from 0 (MRD1 =  >4 mm) to 4 (MRD1 =  <1 mm). I scores ranged from 0 (MRD2  =  ≥2–3 mm) to 4 (MRD2 =  >6–7 mm). To measure levator muscle function the patient's eyebrow was stabilized by pressure exerted with the examiner's thumb. The patient was requested to look fully up, then fully down, while the excursion of the eyelid was measured against a ruler. L scores ranged from 0 to 4 (0 =  ≥14 mm levator muscle excursion; 4 =  ≤5 mm excursion). Clinical examination did not include exophthalmometry because all enrollees using PGA administered drops in both eyes. **Table 1** shows the range of measurements for MRD 1, MRD 2, and levator muscle excursion that are based on a standard measurement protocol used in another study and used to assign P, I and L scores respectively [12].

**Table 1:** Grading Scheme for Marginal Reflex Distance 1, Marginal Reflex Distance 2 and Levator Muscle Excursion.

Ocular Adnexal Anatomy Grade	0 (normal)	1 (good)	2 (fair)	3 (poor)	4 (very poor)
Marginal Reflex Distance1 (mm)	≥ 4	> 3	≥ 2	≥ 1	< 1
Marginal Reflex Distance 2 (mm)	≥ 2–3	> 3–4	> 4–5	> 5–6	> 6–7
Levator Muscle Excursion (mm)	≥ 14	≥ 12–13	≥ 9–11	≥ 6–8	≥ 5

Ptosis of upper eyelid is measured with Marginal Reflex Distance 1 and used to assign a P or ptosis score. Marginal reflex distance 2 values were used to assign an I or inferior scleral show score. Levator muscle excursion values were used to assign an L score.

### Data extracted from the medical record

We extracted patient demographic data, past medical history, and ocular history from the medical record. We obtained a detailed ocular drug history on each subject using both medical records and by personal interview. Patient demographic data included age, sex, and ethnicity. Past medical history included diagnoses of hypertension and diabetes. Past ocular history included glaucoma diagnosis, sub-classified into primary open angle glaucoma, normal tension glaucoma, ocular hypertension, open-angle glaucoma suspect, exfoliation glaucoma, pigmentary glaucoma, and chronic angle closure glaucoma. We also assessed past ocular history for evidence of age-related macular degeneration (AMD), prior intraocular laser surgery such as a laser trabeculoplasty, or laser iridotomy, and childhood strabismus surgery.

Glaucoma drug use was categorized as being current, past, or never according to each class of medicine (PGA, beta blocker, carbonic anhydrase inhibitor, alpha agonist and cholinergic agent). PGA use was further defined by whether it was latanoprost, travoprost or bimatoprost (either 0.01% or 0.03%). Finally, duration of current and time since past glaucoma drug use was rounded up to the highest month.

### Statistical analyses

We performed three interim assessments of interobserver agreement in digital photograph grading of D and S scores to rule out reader drift during the course of the study using the kappa statistic. Interobserver agreement for D and S was found to be high for both eyes (κ≥0.73) based on benchmarks proposed for reliability of categorical data in previous studies [13]. *Intraobserver* agreement in photograph grading of D and S over time was assessed at the midpoint of data collection with the kappa statistic and was also found to be high for both eyes (κ = 0.70) [13]. Correlation between clinical and photographic D (R^2^≥0.86) and S (R^2^≥0.71) scores was calculated with Pearson's correlation coefficient and was found to be moderate to high in both eyes [13].

Unpaired t-tests assessed the relation between current PGA use and adnexal features. The right and left eyes were analyzed separately. Subsequently, we performed ordinal logistic regression analyses accounting for multiple covariates to assess the relation between PGA use and scores for adnexal features. Covariates accounted for in our analyses included age, sex, and ethnicity; body mass index (kilograms/meter^2^); diagnosis of hypertension, diabetes, glaucoma, and AMD; prior intraocular laser surgery; and current use of non-PGA medications for the treatment of glaucoma. We controlled for each of the following categories of non-PGA medication use individually: topical beta-blockers, alpha-adrenergic agonists, topical and oral carbonic anhydrase inhibitors. Only a few patients were on cholinergic medications so this variable was not included in multivariable analysis. We assessed the relation between *current* use of any PGA and D, P, L, S, and I scores. Subsequently we used current use of specific PGAs as the exposures of interest in relation to the adnexal features. Too few patients were using bimatoprost 0.01% for any meaningful assessment and these patients were excluded from multivariable analyses. We also analyzed the association of *duration* of use of any PGA and *past* use of any PGA in relation to D, P, L, S, and I scores. Finally, we repeated analyses for duration of use of bimatoprost, travoprost, and latanoprost as the exposure of interest in relation to adnexal features.

We used SAS (v9.2, SAS Institute, Cary, NC) for all statistical analyses. The nine exposures in our analysis included: current PGA use, current bimatoprost 0.03% use, current travoprost use, current latanoprost use, past PGA use, duration of PGA use, duration of bimatoprost use, duration of travoprost use, and duration of latanoprost use. The five outcomes in our analysis included digital D, digital S, clinical P, clinical L, and clinical I scores. We corrected for multiple comparisons using the Bonferroni correction as detailed in the footnotes of relevant tables in the results section. P values considered significant are less than or equal to 1.11E-03.

## Results

The study population consisted of Caucasian (73%), African American (18%), Hispanic (5%), and Asian patients (3%) with a mean age of 65.4 ± 9.4 years. The distribution of basic demographic features and medical status stratified by PGA use is provided in **Table 2** **.** Any imbalance in covariates between PGA users and non-users was adjusted for in ordinal logistic regression analysis. 269 (78%) patients reported a diagnosis of glaucoma that was confirmed by analysis of medical records. 92 (27%) patients underwent ocular laser surgery such as argon laser trabeculoplasty, selective laser trabeculoplasty, and laser peripheral iridotomy prior to participation in the study.

**Table 2:** Characteristics of Study Participants.

	Current Latanoprost 0.005% User (n = 64)	Current Travoprost 0.004% User (n = 43)	Current Bimatoprost 0.03% User (n = 50)	Current PGA Nonuser (n = 186)
Age (years)	65.7 ± 9.7	65.9 ± 9.8	65.1 ± 9.1	65.3 ± 9.3
Ethnicity				
Caucasian, %	68	69	60	79
African American, %	24	25	26	12
Hispanic, %	0	6	10	6
Asian, %	8	0	4	3
Female,%	45	54	53	59
Diabetes mellitus, %	17	23	25	15
Hypertension, %	63	47	68	50
BMI in kilogram/meter^2^	26.0 ± 4.4	26.1 ± 3.9	27.5 ± 5.5	26.2 ± 4.7
Glaucoma, %*	97	98	98	63
Age related macular degeneration, %*	6	9	1	3
Laser surgery, %*	39	20	42	21
Current topical beta blocker use, %*	50	51	53	19
Current oral or topical CAI use, %*	46	41	57	11
Current AA use, %*	37	23	38	7
Current cholinergic use, %*	1	0	2	0

Values are means ± standard deviation or percentages and are standardized to the age distribution of the study population. *The above parameters refer to the right eye. Results were similar for the left eye and are not shown. Abbreviations used: PGA =  prostaglandin analogue; STD = standard deviation; BMI =  body mass index;

CAI =  carbonic anhydrase inhibitor; AA = alpha agonist

Of 157 patients undergoing treatment with PGAs, 50 patients were currently using bimatoprost, 43 were using travoprost, and 64 were using latanoprost. Mean duration of current PGA use was 18 months for both eyes and 8% of participants were past users of PGAs.

### Relation of Current Prostaglandin Analogue Use with Ocular Adnexal Features

Analyses were limited to the right eye as data between eyes was similar and highly correlated. In multivariable analysis current PGA use was associated with a 230-fold increased risk of a 1-unit increase in D score (odd ratio (OR)  =  2.30; 95% confidence interval (CI), 1.43, 3.69) based on masked assessment of digital external photographs signifying an association with less dermatochalasis or DUES (**Table 3**). The result was highly correlated with clinical D scores (R^2^≥0.86). Current use of bimatoprost 0.03% (OR  =  3.67; 95% CI, 1.82, 7.40) and current use of travoprost (OR  =  3.43; 95% CI, 1.70, 6.91) were most strongly associated with higher D score while current latanoprost use (OR  =  1.42; 95% CI, 0.79, 2.57) was not significantly associated with D score, even though the latter agent was well represented in terms of percent and duration of use (mean duration 10 months) in this study sample.

**Table 3:** Multivariable odds ratio (95% confidence interval; p-value) for current prostaglandin analogue (PGA) use associated with selected ocular adnexal features.

	Any PGA Use (n = 341)	Bimatoprost 0.03% Use (n = 232)*	Travoprost 0.004% Use (n = 228)**	Latanoprost 0.005% Use (n = 248)***
Digital D Score	2.30 (1.43, 3.69); **p = 5.44E-04**	3.67 (1.82, 7.40); **p = 2.75E-04**	3.43 (1.70, 6.91); **p = 5.54E-04**	1.42 (0.79, 2.57); p = 0.24
Digital S Score	2.49 (1.54, 4.03); **p = 1.98E-04**	8.35 (3.94, 17.69); **p = 3.06E-08**	2.18 (1.08, 4.41); p = 0.03	1.78 (0.97, 3.25); p = 0.06
Clinical P Score	4.04 (2.43, 6.72); **p = 7.37E-08**	5.04 (2.41, 10.56); **p = 1.80E-05**	5.10 (2.42, 10.76); **p = 1.91E-05**	3.53 (1.87, 6.66); **p = 9.80E-05**
Clinical L Score	7.51 (3.39,16.65); **p = 6.74E-07**	14.90 (5.31, 41.79); **p = 2.84E-07**	8.65 (2.92, 25.68); **p = 1.01E-04**	5.25 (1.98, 13.96); **p = 8.83E-04**
Clinical I Score	2.60 (1.58, 4.28); **p = 1.72E-04**	2.75 (1.33, 5.69); p = 0.01	2.90 (1.40, 6.01); p = 4.27E-03	2.22 (1.19, 4.13); p = 0.01

The reported odds ratios (ORs) reflect likelihood of increasing scores. Increasing digital D score reflects loss of upper lid dermatochalasis judged from digital photos. Increasing digital S score reflects loss of lower lid steatoblepharon judged from digital photos. Increasing clinical P score reflects increasing upper lid ptosis as judged by a masked clinical observer. Increasing clinical L score reflects worsening levator function as judged by a masked clinical observer. Increasing clinical I score reflects increasing inferior scleral show as judged by a masked clinical observer.

• Data is for the right eye. Digital scores reflect mean of 2 readers.

• Bonferroni correction calculation: 0.05/9 exposures x 5 outcomes =  1.11E-03. p-values that are significant after Bonferroni correction are in bold.

• ORs are corrected for age, ethnicity, sex, use of other glaucoma medications, ocular disease such as age-related macular degeneration and glaucoma, systemic disease such as hypertension and diabetes, and history of ocular laser surgery using ordinal logistic regression.

* Patients who were current travoprost users and current latanoprost users were excluded from this analysis. ** Patients who were current bimatoprost users and current latanoprost users were excluded from this analysis. *** Patients who were current bimatoprost users and current travoprost users were excluded from this analysis.

Current PGA use was also associated with a 249-fold increased risk of a 1-unit increase in S score (OR  =  2.49; 95% CI, 1.54, 4.03) based on assessment of digital external photographs signifying an association with loss of inferior periorbital fat pads (**Table 3**). The association was strongest with bimatoprost 0.03% use (OR  =  8.35; 95% CI, 3.94, 17.69). Similar trends were seen for travoprost and latanoprost use but they were not significant after accounting for the multiple comparisons made. Similar results were seen between current PGA use and clinical S score (OR  =  4.23; 95% CI, 2.51, 7.13).

Current PGA use was strongly associated with increased risk of upper eyelid ptosis as measured by clinical P score (OR  =  4.04; 95% CI, 2.43, 6.72) and increased risk of upper lid levator dysfunction as measured by clinical L score (OR  =  7.51; 95% CI, 3.39, 16.65) (**Table 3**). All PGAs contributed significantly to this association. Current PGA use was also associated with a 260-fold increased risk of a 1-unit increase in I score (OR  =  2.60; 95% CI, 1.58, 4.28) signifying an association with lower lid retraction. No specific PGA showed a particularly strong association with increasing I score.

### Relation of Duration of Prostaglandin Use with Ocular Adnexal Features

Only longer duration of bimatoprost 0.03% use was significantly associated with higher D (OR  = 1.03; 95% CI, 1.01–1.04), S (OR  =  1.04; 95% CI, 1.02–1.06), and L (OR  = 1.03; 95% CI, 1.01–1.05) scores (Table 4). Thus each 1-month of bimatoprost 0.03% use was associated with a 3%–4% chance of an incremental increase in D, S, and P scores. Longer duration of travoprost or latanoprost use was not associated with significantly higher scores in any category of ocular adnexal features analyzed (**Table 4**).

**Table 4:** Multivariable odds ratio (95% confidence interval; p-value) for duration of current prostaglandin analogue (PGA) use in months associated with selected ocular adnexal features.

	Duration Any PGA Use (n = 335)	Duration of Bimatoprost 0.03% Use (n = 232)*	Duration of Travoprost 0.004% Use (n = 227)**	Duration of Latanoprost 0.005% Use (n = 243)***
Digital D Score	1.01 (1.00, 1.01); p = 0.06	1.03 (1.01, 1.04); **p = 3.85E-04**	1.01 (1.00, 1.03); p = 0.07	1.00 (1.00, 1.01); p = 0.33
Digital S Score	1.01 (1.00, 1.02); p = 0.01	1.04 (1.02, 1.06); **p = 2.25E-05**	1.00 (0.99, 1.02); p = 0.78	1.01(1.00, 1.02); p = 0.01
Clinical P Score	1.01 (1.00, 1.02); p = 1.54E-03	1.02 (1.01, 1.04); p = 2.96E-03	1.02 (1.01, 1.04); p = 4.02E-03	1.01 (1.00, 1.02); p = 0.01
Clinical L Score	1.01 (1.00, 1.01); p = 0.16	1.03 (1.01, 1.05); **p = 5.24E-04**	1.03 (1.01, 1.05); p = 0.01	1.00 (0.99, 1.02); p = 0.56
Clinical I Score	1.01 (1.00, 1.02); p = 0.04	1.01 (1.00, 1.03); p = 0.11	1.02 (1.01, 1.04); p = 0.01	1.01(1.00,1.02); p = 0.17

The reported odds ratios (ORs) reflect likelihood of increasing scores. Increasing digital D score reflects loss of upper lid dermatochalasis judged from digital photos. Increasing digital S score reflects loss of lower lid steatoblepharon judged from digital photos. Increasing clinical P score reflects increasing upper lid ptosis as judged by a masked clinical observer. Increasing clinical L score reflects worsening levator function as judged by a masked clinical observer. Increasing clinical I score reflects increasing inferior scleral show as judged by a masked clinical observer.

• Data is for right eye. Digital scores reflect mean of 2 readers.

• Bonferroni correction calculation: 0.05/9 exposures×5 outcomes =  1.11E-03. p-values that are significant after Bonferroni correction are in bold.

• ORs are corrected for age, ethnicity, sex, use of other glaucoma medications, ocular disease such as age-related macular degeneration and glaucoma, systemic disease such as hypertension and diabetes, and history of ocular laser surgery using ordinal logistic regression.

* Patients who were current travoprost users and current latanoprost users were excluded from this analysis. ** Patients who were current bimatoprost users and current latanoprost users were excluded from this analysis. *** Patients who were current bimatoprost users and current travoprost users were excluded from this analysis.

### Past Use of Prostaglandin with Ocular Adnexal Features

Past use of any PGA was not associated with significantly higher scores in any adnexal category (p = 0.26). Mean duration since use of any PGA was 42.8 ± 34.9 months.

## Discussion

This large prospective, cross-sectional study showed associations between current bilateral PGA use and deepened upper eyelid sulci, hollowing of the inferior periorbital fat pads, upper eyelid ptosis with levator muscle dysfunction, and lower lid retraction, although the latter finding was not particularly robust. These findings are strongly driven by use of bimatoprost 0.03%, a formulation that is no longer marketed in the United States on the Allergan website (see www.lumigan.com). Although previous studies reported loss of dermatochalasis and inferior orbital fat pads, the findings of ptosis and levator muscle dysfunction associated with use of all members of the PGA class have not been previously emphasized. Interestingly, all the PGA manufacturers include a postmarketing statement regarding deepening of the upper lid sulci but make no mention of loss of lower eyelid steatoblepharon, levator dysfunction and upper eyelid ptosis [14], [15], [16]. This is the first study to account for multiple potential confounders in assessing the relation between bilateral PGA use and adnexal features.

The mechanism by which PGAs produce the deepening of the upper eyelid sulcus and loss of inferior orbital fat pads seems to involve effects on periorbital adipocytes as first suggested by Filippopoulous et al [5]. FP-prostanoid receptor activation inhibits preadipocyte differentiation in several cell lines that are then prevented from expressing adipocyte-specific genes [5]. Preaponeurotic fat biopsies from patients using the various PGAs and controls demonstrated that mean adipocyte density of bimatoprost and travoprost treated eyes was significantly higher (p<0.001) than that of untreated eyes indicating the volume of lipid per cell was reduced in PGA users [17]. Jayaprakasam and Ghazi-Nouri provide *in situ* neuroradiologic confirmation of periorbital fat atrophy in bimatoprost and travoprost users [18].

The mechanism of ptosis and levator muscle dysfunction in PGA users is unknown. One mechanism may involve chemical dehiscence of the levator muscle from the superior tarsal plate. In a retrospective study of floppy eyelid syndrome, immunohistochemistry of eyelid biopsy specimens demonstrated increased immunoreactivity for elastolytic proteases, particularly matrix metalloproteinase (MMP) 7 and MMP 9 [19]. Weinreb et al. demonstrated that prostaglandin F2α exposure increased production of MMP 1, MMP 2, MMP 3, and MMP 9 by ciliary body smooth muscle cells [20]. Furthermore, Ooi et al. treated isolated human ciliary body smooth muscle cells with free acid forms of bimatoprost, latanoprost, and unoprostane and found that these PGAs increased concentrations of MMP-1, MMP-3, and MMP-9 [21]. A contrary view and one that fits with our clinical impression is that low grade dermatitis, erythema, and hyperpigmentation of the lid tissue produces lid tightening which in turn makes the lid stiff and ptotic.

Lower eyelid retraction was the least prominent effect we observed with PGA use (OR  =  2.60; 95% CI, 1.58, 4.28) and probably needs to be studied with a larger sample size. Nonetheless we speculate that the modest lower lid retraction we observed is related to contraction of smooth muscle in the lower eyelid. There is evidence that the PGAs, especially bimatoprost, are potent agonists in stimulating contraction of human ciliary smooth muscle although, admittedly similar studies of eyelid smooth muscle have not been performed [22].

Our results are consistent with Aihara et al. [23] who performed the first prospective study of the incidence of DUES after use of bimatoprost in twenty-five open-angle glaucoma patients. Patients were switched from latanoprost to bimatoprost and observed over 6 months with external photographs. At 3 and at 6 months, 60% (15 out of 25) of the patients had DUES as judged photographically by three examiners [23]. Specifically, the study by Aihara et al. and our study both show that prostaglandin associated periorbitopathy (PAP) is pervasive as indicated by the high incidence rate in their study and the high odds ratio we found for DUES in current bimatoprost users (OR  =  3.67; 95% CI, 1.82, 7.40). Another study by Inoue et al. also found DUES to be most common among bimatoprost users [24]. The effect seems to develop soon after starting treatment with bimatoprost 0.03%. For example we found that the odds ratio for an incremental increase in D score for current bimatoprost 0.03% users was 3% per month of use (OR  =  1.03; 95% CI, 1.01–1.04). Interestingly we did not see a significant duration effect for chronic travoprost or chronic latanoprost use, perhaps because our sample size may not have been adequate and/or because these latter agents require longer time to produce adnexal tissue changes.

Overall, our study has several strengths. To our knowledge, this is the largest, prospective study of the association between bilateral PGA use and select ocular adnexal features performed thus far. The grading scheme we constructed to assess digital photographic D and S was validated with high interobserver agreement prior to the start of data collection and digital grading. Both clinical examiners and digital photograph readers were masked to glaucoma diagnosis and history of PGA use in study participants. Analyses were adjusted for many key covariates, including age, gender, ancestry, body mass index (BMI), the use of glaucoma medications other than PGAs, and glaucoma diagnosis. We were able to demonstrate that the associations we studied were strongest with bimatoprost 0.03%, a formulation that has a higher concentration than the other PGAs. This is consistent with the results of preaponeurotic fat pad biopsies performed by Park et al. that showed significantly higher fat cell densities in bimatoprost users [17]. Furthermore, we showed that longer duration of use of bimatoprost is associated with higher D, S, and L scores. This is important because even if our digital readers and clinical examiners unknowingly unmasked themselves by detecting other signs of PGA use (e.g., hypertrichosis, periorbital skin pigmentation), they could not have known about the duration of PGA use.

One limitation of this study is that we could not recruit sufficient number of bimatoprost 0.01% users to perform meaningful analyses. Preliminary studies indicate that bimatoprost 0.01% has acceptable IOP efficacy and as bimatoprost 0.03% users are converted to the lower dose, it will be important to assess the effect of bimatoprost 0.01% on adnexal features [25], [26]. Furthermore, because the study is cross-sectional, we were not able to analyze the reversibility of ocular adnexal changes. Finally the cross-sectional nature of study does not give an accurate estimate for the attack rate for PAP. Despite these caveats, the high odds ratios and significant p-values after Bonferroni correction for multiple comparisons speak to the biological relevance of the correlations we found and suggest that PAP is common.

These observations regarding PAP raise several concerns about the medical management of patients diagnosed with glaucoma. With regard to patients newly starting anti-glaucoma eye medications, it is beneficial to inform patients of ocular adnexal changes that can occur with PGA use. We advise caution before starting PGAs unilaterally because of the potential to induce asymmetry in ocular adnexal anatomy. When managing glaucoma in current PGA users, if the PGA is not adequately lowering IOP, it is reasonable to replace it with a non-PGA anti-glaucoma medication. Finally the risks of continued use of PGAs should be carefully weighed in those patients with inferior visual field loss as the later development of upper eyelid ptosis could compromise visual function.

In conclusion, this work demonstrates that PAP is fairly common and consists of findings that extend beyond DUES to include loss of lower lid steatoblepharon, upper lid levator dysfunction and upper lid ptosis. We suspect that PGA-associated upper lid ptosis is not reversible but more study is needed on this subject. Latanoprost use produced the least amount of periorbital fat loss but it too was associated with upper lid ptosis. More longitudinal analysis of PGA users is indicated.
